# When liquid-liquid phase separation meets viral infections

**DOI:** 10.3389/fimmu.2022.985622

**Published:** 2022-08-09

**Authors:** Wenqiang Wei, Lu Bai, Bing Yan, Weiquan Meng, Hongju Wang, Jingbo Zhai, Fusheng Si, Chunfu Zheng

**Affiliations:** ^1^ Kaifeng Key Laboratory of Infection and Biological Safety, School of Basic Medical Sciences, Henan University, Kaifeng, China; ^2^ Department of Immunology, School of Basic Medical Sciences, Fujian Medical University, Fuzhou, China; ^3^ Medical College, Inner Mongolia Minzu University, Tongliao, China; ^4^ Key Laboratory of Zoonose Prevention and Control at Universities of Inner Mongolia Autonomous Region, Tongliao, China; ^5^ Institute of Animal Science and Veterinary Medicine, Shanghai Key Laboratory of Agricultural Genetics and Breeding, Shanghai Engineering Research Center of Breeding Pig, Shanghai Academy of Agricultural Sciences, Shanghai, China; ^6^ Department of Microbiology, Immunology and Infectious Diseases, University of Calgary, Calgary, AB, Canada

**Keywords:** liquid-liquid phase separation, membraneless organelle, inclusion, viral infection, immune regulation

## Abstract

Eukaryotic cells have both membranous and membraneless organelles. While the formation mechanism of membranous organelles is well understood, the formation mechanism of membraneless organelles remains unknown. Many biomolecules in the cytoplasm transition from the liquid phase to the agglutinated phase are known as liquid-liquid phase separation (LLPS). The biomolecular agglomerates’ physical properties enable them to function as dynamic compartments that respond to external pressures and stimuli. Scientists have gradually recognized the importance of phase separation during viral infections. LLPS provides a powerful new framework for understanding the viral life cycle from viral replication to evasion of host immune surveillance. As a result, this review focuses on the progress of LLPS research in viral infection and immune regulation to provide clues for antiviral therapeutic strategies.

## Background

The regulation, coordination, and networking of different cellular compartments underlie the function of biological systems. Among these compartments are membrane and non-membrane organelles. The membrane-bound organelles carry out functions in a selective and specific manner without any external disturbance. The exchanging information between membrane organelles is endorsed by mechanisms such as fusion and fission and vesicles trafficking in the endomembrane system (1). However, large gaps remain in our understanding of the collaboration and regulation of membraneless organelles (MLOs) in biochemical functions.

Recent studies have shown that macromolecules’ liquid-liquid phase separation (LLPS) may be the physicochemical basis for forming non-membrane organelles inside the cells (2–4). In the compartments formed by LLPS, specific molecules are concentrated in fluid-like liquid droplets that coexist stably with the surrounding fluid environment. Some examples of these biomolecular condensates include processing bodies (P-bodies), stress granules (SGs), Cajal bodies, Nucleosomes, nuclear speckles, membrane clusters, signaling puncta, Germ granules, Balbiani bodies, paraspeckles, DNA damage foci, histone locus, viral replication compartments (RCs) and inclusion bodies (IBs) (3, 5–11). After the formation of LLPS, the biomolecule exists in two forms, one at low concentration in bulk dilute phase and one at higher concentration in the formed “droplets”. The polymer molecules usually move within the dense phase or between the dense and bulk dilute phases (12, 13). The interconversion of these two phases depends upon the change in the surrounding cellular environment.

The occurrence of LLPS is highly dependent on the concentration of biomolecules (proteins, DNA, and RNA) in the solution, their physicochemical properties, and the solution environment (temperature, pH, salt concentration, and salt ion type) (14, 15). The threshold concentration of biomolecules is the major factor contributing to the phase separation of homogeneous solutions. When the concentration of biomolecules exceeds the threshold concentration, they begin to aggregate, leading to the appearance of LLPS (4). The other influencing factors include chaperones, ATP, post-transcriptional modification, pH, ionic strength, and temperature (10). Furthermore, various intermolecular interactions, including ionic bonds, van der Waals forces, hydrogen bonds, π-π, and π-cation of aromatic residue and cation amino acid residue, are also involved in the occurrence of phase separation (16–19).

Viruses are obligate intracellular parasites that rely on the host machinery for viral replication. The concept of phase separation provides new insights into understanding the mechanisms of viral infection. Several studies revealed that viral infection is associated with membraneless condensates (9). Some viruses have been shown to assemble biomolecular condensates with liquid properties, such as rabies virus (RABV), vesicular stomatitis virus (VSV), and severe acute respiratory syndrome coronavirus 2 (SARS-COV-2) (20–22). Moreover, these condensates are also associated with SGs, suggesting the condensate’s potential roles in the innate immune responses (23). A growing body of studies reveals the important role of LLPS in the viral life cycle, including viral entry, genome replication, assembly, and viral packaging, as well as antiviral innate immune signaling (20, 23). In this review, we will focus on the role of LLPS in viral infection and immune regulation to provide a novel insight into antiviral therapeutic strategies.

## Cellular factors that drive phase separation

Studies have revealed that several factors, including multivalency of proteins, temperature, ionic strength, RNA elements, and metal ions, contribute to forming liquid droplets (17). Here, we introduced these factors’ roles in forming LLPS.

### Intrinsically disordered regions

Intrinsically disordered regions (IDRs) of a protein have no specific three-dimensional structure and can weakly and multivalently interact with other proteins, resulting in liquid condensates (11, 15, 24). Numerous studies have shown that weak and multivalent RNA-protein or IDR-IDR interactions are critical for the high-order assembly of biomolecules (25, 26). For example, IDRs in transcriptional coactivators BRD4 and MED1 are integral for driving phase separation (27, 28). Besides this, prion-like domains (PLDs), similar to low-complexity sequence domains (LCDs), can also drive LLPS *in vivo* (29). The valence of aromatic residues in PLDs plays a major role in LLPS, and a specific sequence of aromatic residues helps form liquid droplets. Moreover, intrinsically disordered proteins (IDPs) are also subject to phase separation resulting in the formation of membraneless organelles with various cellular functions (30). IDPs exhibit a high conformational heterogeneity due to lacking a stable and precise secondary or tertiary structure (31). Polar charged residues promote the formation of disordered proteins; therefore, IDPs are also considered polyelectrolytes (32). The protein IDRs are indispensable for forming membraneless organelles through LLPS.

### Protein multivalency

The multivalency of protein contributes to phase separation. For example, mixing an engineered protein containing multiple SRC homology 3 (SH3) repeats and another containing multiple proline-rich motifs (PRM) repeats resulted in phase separation *in vitro* (12). Another example is the nephrin-Nck-N-WASP system, in which phosphorylated nephrin binds to the SH2 domains of Nck while three SH3 domains of Nck can further bind to N-WASP six PRMs. The multivalency of these proteins results in phase separation (33). Besides SH3, other multidomain modules are also involved in phase separation. For instance, a coiled-coil trimer formed by SynGAP can bind to multiple copies of PSD-95, leading to the formation of LLPS (34). Altogether, the formation of multimers mediated by multivalent interactions can drive the formation of LLPS.

### Genomic RNA elements

Like specific multivalent protein-binding sites, Genomic RNA elements are indispensable for phase separation. It has been found that the phase separation mediated by IDP and RNA is RNA concentration-dependent. A low amount of RNA promotes phase separation, while a high amount can inhibit it (35). A recent study showed that distinct regions of viral genomic RNA have distinct roles in mediating phase separation (36). For instance, the nucleocapsid encoding region located at the 3′end of severe acute respiratory syndrome coronavirus (SARS-CoV) genomic RNA (gRNA) can promote phase separation while its frameshifting and packaging signal region can dissolve the liquid phase (37). Another study found that the viral gRNAs bind to the IDRs and RNA-binding domains of the N protein to mediate phase separation, promoting the assembly of virus particles (38).

### Zn^2+^ ions

The metal ions regulate phase separation and are related to developing some diseases (39–41). It has been found that Zn^2+^, but not other ions (Mn^2+^, Cu^2+^, and Fe^2+)^, plays a significant role in the phase separation of tau protein (42). The multiple zinc-binding sites of tau are required for the formation of LLPS. Metal ions can react with prion-like disordered protein domains (PrLDs), providing us with a doctrine to further understand phase separation (43). Further study about the mechanism of tau-mediated phase separation may improve the treatment of tau-associated degenerative diseases (44). Moreover, Zn^2+^ is also involved in the virus-mediated phase separation. For instance, human immunodeficiency virus type 1 (HIV-1) nucleocapsid proteins are required for zinc finger (ZnF) protein-dependent LLPS, regulating genomic RNA positioning and trafficking (45). The ZnF NC mutant and Zn2+ chelation inhibited NC co-localization with vRNA and suppressed NC-mediated LLPS (45–47). RABV and VSV can employ Zn^2+^ to regulate the formation of liquid condense mediated by N-protein and nucleic acid, promoting virus assembly (48). It is interesting to study whether the inhibition of ZnF protein can suppress viral replication.

## Roles of virus-driven phase separation during virus infection

Because the viral replication cycle is highly dependent on the infected host cell, viruses have evolved to utilize and remodel cellular structures to facilitate viral replication and counteract host cell resistance to viral infections.

**Figure 1:**
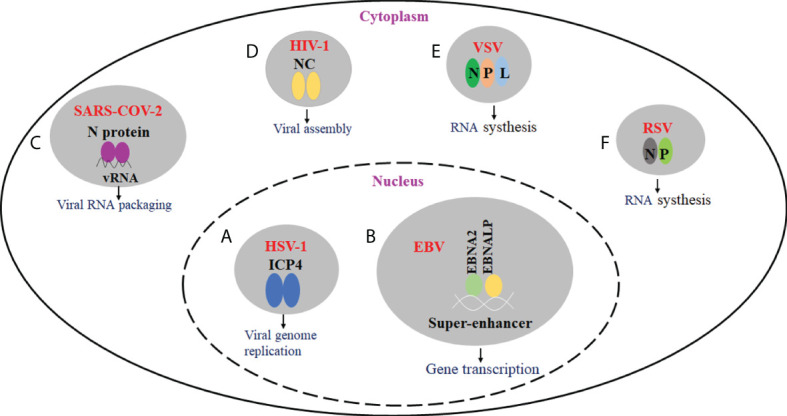
The effect of virus-driven phase separation on the viral infection. **(A)** HSV-1 ICP4 can drive the formation of condensates in the nucleus *via* LLPS. ICP4 is localized to RCs and required for viral replication. **(B)** EBV EBNA2, EBNALP, and other transcription factors could form condensate at super-enhancers *via* LLPS. They may be involved in the epigenetic regulation of chromatin activation. **(C)** SARS-CoV-2 N protein can undergo LLPS in the presence of viral RNA, and LLPS may be implicated in the assembly of progeny virions. **(D)** For HIV-1, nucleocapsid (NC)-mediated LLPS induces translational silencing and drives viral assembly. **(E)** IBs are proved to be membraneless organelles. For VSV, viral IBs were formed in the presence of N, P, and L proteins, and viral RNA synthesis occurs in IBs. **(F)** For RSV, viral IBs were formed in the presence of N and P proteins, which play an important role in viral RNA synthesis.

Many viruses have been shown to produce biomolecular condensates with liquid properties. The formation of biomolecular agglutinates substantially leads to a significant increase in local molecular concentration and intermolecular contacts, thus enhancing the rate of biochemical reactions. Further study showed that biomolecular condensates play a role in viral genome replication, transcriptional translation, nucleocapsid assembly, and egress. For example, DNA viruses form nuclear viral replication compartments through phase separation, and many negative RNA viruses induce the formation of viral IBs (49, 50). Besides, by sequestering antiviral sensors into viral IBs, biomolecular condensates can also prevent the activation of innate immune pathways (51–54). Here, we will cover recent progress on the roles of biomolecular condensates in the viral infection process and immune response (**Figure 1**).

### Phase separation regulates virus replication

#### The roles of phase separation in DNA virus replication and transcription

The action of phase separation in virus replication and transcription has been reported in some members of the Herpesviridae family. It is generally accepted that the DNA replication, gene transcription, and nucleocapsid assembly of herpes simplex virus type 1 (HSV-1), a member of the alpha-Herpesviridae family, occur in the nucleus of host cells (55). When the viral genome enters the nucleus, HSV-1 sequentially expresses immediate-early, early, and late proteins. The immediate-early proteins activate the transcription and translation of early genes, which participate in the viral genome replication and replication compartments (49, 56, 57). Recent studies have shown that the immediate-early protein ICP4 is an intrinsically disordered protein that can drive the formation of condensates in the nucleus *via* LLPS (58). Since ICP4 is required for viral replication and is localized to RCs, RCs may be the product of LLPS (59).

It should be noted that not all the proteins in RC are recruited through LLPS. For example, the recruitment and retention of RNA polymerase II (Pol II) in the RC is achieved *via* the non-specific binding of Pol II to HSV-1 DNA (60). Therefore, the formation mechanisms of RCs might be complex, and the roles of LLPS in RC compartmentalization need further investigation. After completing capsid assembly and genome packaging in the nucleus, HSV-1 nucleocapsid undergoes primary envelopment and de-envelopment at the nuclear envelope, followed by secondary envelopment in the cytoplasm (55, 61). Tegument protein acts as a link between the capsid and the viral envelope, promoting secondary encapsulation. A recent study found that HSV-1 tegument protein UL11 possesses the IDR and can form LLPS *in vitro* (62). Interestingly, it is also found that several HSV-1 tegument proteins have IDRs and might have the potential to undergo LLPS, suggesting that the tegument proteins might play a role in tegument assembly through LLPS.

The first identified human oncovirus is Epstein Barr virus (EBV), belonging to the gamma subfamily of the Herpesviridae family. EBV is closely related to multiple malignant tumors, including nasopharyngeal carcinoma and gastric cancer (63, 64). EBNA2 and EBNALP are two EBV-encoded transcription factors expressed early after EBV infection in the B cells (65). Co-expression of both proteins can drive quiescent B cells into the cell cycle and promote B cell transformation (66). Importantly, EBNA2 binding sites are positioned near the promoter and enhancer elements in viral and cellular genomes. Recent studies found that EBNA2 can be enriched in super-enhancer regions formed by many transcriptional enhancers (67–69).

Moreover, EBNA2, EBNALP, and other transcription factors could form condensate at super-enhancers *via* LLPS (70). Furthermore, EBNA2 can remodel chromatin topology through phase separation, resulting in the formation of accessible chromatin domains (ACDs) in the host genome. The N-terminal of EBNA2 is required for ACD induction and phase separation formation, whereas the C-terminal can recruit histone acetyltransferase p300 to ACDs for acetylation of ACDs (71). As a result, phase segregation theoretically supports further epigenetic regulation of chromatin activation and genomic transcription.

#### Phase separation regulates RNA virus replication

The effects of phase separation on virus replication were well explored in the N protein of SARS-CoV-2. The genomes of SARS-CoV-2 are encapsulated by N protein. The presence of several RNA binding domains in this nucleocapsid protein, including low-complexity areas and oligomerization domains, suggests that N protein can create biomolecular condensates (72). It has been demonstrated that N proteins can undergo LLPS *in vitro*. The turbidity experiments revealed that increased RNA concentration would enhance the turbidity of droplets (23). However, the turbidity decreases when RNA concentration exceeds a certain threshold due to the classical reentrant behavior (73). By measuring turbidity and spherical droplet size at different NaCl concentrations, it is found that electrostatic interactions inhibit the formation of LLPS mediated by N proteins and RNA (38). The LINK region of N protein contains a serine- and arginine-rich SR region. The phosphorylation of the SR region leads to the formation of salt bridges between the phosphate groups and arginine side chains, inhibiting N-protein and RNA-induced LLPS (23).

The N protein-mediated LLPS is a multifunctional protein involved in multiple infection processes. The SARS-CoV-2 requires RNA-dependent RNA polymerase (RdRp) and a series of cofactors during replication and transcription (74). Fluorescence co-localization experiments demonstrate that N proteins recruit RdRp/RNA complexes and promote virus replication. LLPS may promote ribonucleoprotein condensate formation while packaging viral RNA genomes into nascent virions (72). It is speculated that newly synthesized N proteins form pre-capsids with viral genomes *via* LLPS. Then the pre-capsids are released upon maturation and interact with structural proteins in the ER-Golgi intermediate compartment (ERGIC) for subsequent packaging (74–77).

In addition, various experiments have shown that N proteins can regulate the formation and function of SGs. SG formation can inhibit protein synthesis, limit energy consumption, repair stress-induced damage, and promote cell survival (78). However, SARS-CoV-2 can phosphorylate eIF^2+^ by activating the kinases PKR and PERK, which induce SG formation (79). However, SG formation does not inhibit viral proliferation, indicating that SARS-CoV-2 can counteract host cell responses (80). It has been shown that the stress granule assembly factors 1 and 2 (G3BP1/2) can interact with N proteins (81). Perdikari et al. further found that N proteins can partition into liquid phases formed by hnRNPA2, FUS, and TDP-43, demonstrating that N proteins can interact with many particle-associated heterogeneous nuclear ribonucleoproteins (hnRNPs) (38). Therefore, it is speculated that N proteins, once recruited to SG, may selectively sequester the key components in SG to convert SG into a site for promoting viral replication.

#### Phase separation regulates virus assembly

Here, we take HIV-1 as an example to demonstrate the role of phase separation in regulating virus assembly. HIV-1 has a complex viral life cycle as a retrovirus, including fusion, decapsulation, reverse transcription, and integration into the host cell to form a provirus, transcription of a large amount of positive-stranded RNA before exiting the nucleus. These positive-stranded RNAs can act as mRNAs directing the synthesis of viral proteins and as vRNAs assembled with the viral core proteins to form immature viral particles before budding and becoming mature viral particles (82–84). The balance between mRNA translation and RNA and core protein packaging is regulated by nucleocapsids-mediated LLPS.

HIV-1 Gag polyprotein precursor (also known as Pr55Gag) regulates viral assembly. The Gag is proteolytically sheared into multiple monomeric protein matrices (MA), capsid (CA), nucleocapsid (NC), p6 structural domain, and two spacer peptides SP1 and SP2 (85, 86). NC plays an important role in key cycle processes such as vRNA capsulization and Gag multimerization (87). The typical structural features of NC are two highly conserved CCHC-type zinc finger structures. NC also has a low-complexity, intrinsically disordered prion-like domain (43, 88). Anne Monette et al. showed that HIV-1 NC could form droplets through LLPS. NC-mediated LLPS induces translational silencing and drives viral assembly by affecting the balance between viral RNP and NC-mediated SG, resulting in the formation of infectious viral particles. To avoid viral RNP overgrowth leading to the production of dysfunctional viral particles, the formation of RNP is therefore also limited by NC-mediated SG. It has been shown that overexpression of NC protein leads to the induction of SG assembly (89).

#### Phase separation is involved in the formation of IBs

IBs are proved to be membraneless organelles (90). They play an important role in virus genomic replication and transcription. Like other membraneless organelles, RSV IBs are formed in the presence of N and P through LLPS. In the N-P complex, the C-terminus of P protein interacts with N_NTD_, and its N-terminus interacts with N_CTD_. Furthermore, the oligomerization domain and the C-terminal IDR of P are indispensable for forming IBs (91). VSV can form cytoplasmic inclusions with classical fluidic properties. When the inclusions are formed, RNA synthesis machinery is redistributed to inclusions, where RNA synthesis occurs (90). The cytoplasmic inclusions are membraneless structures. The live-cell fluorescence microscopy found that they are dynamic organelles capable of fission and fusion. In addition, cytoplasmic inclusions can maintain roundness induced by intrinsic surface tension. Moreover, the proteins in the inclusions can reversibly exchange with the cytoplasmic pool (21). Although belonging to the same Rhabdoviridae, VSV partially differs from RABV that formed the classical liquid compartments, Negri bodies (NBs), through LLPS (20, 91). In the case of RABV, the presence of N and P proteins can drive the formation of a very small NB, but for VSV, viral IBs were formed in the presence of N, P, and L proteins (21).

#### Impact of virus-driven phase separation on host antiviral innate immunity

Since the phase-separated region can specifically enrich a protein and its interacting proteins while excluding others, the virus might use this strategy to evade the host’s innate immune responses.

Recent studies have shown that LLPS plays an important regulatory role in the cGAS- stimulator of interferon genes (STING) immune pathway. cGAS is activated by cytoplasmic viral DNA and synthesizes the unique second messenger cyclic GMP- AMP (cGAMP) (92–94). cGAMP binds to the ER-localized junction protein STING and drives the conformational change of STING (95). STING can activate the TBK1-IRF3 pathway, promoting the production of type I IFN (96). The cGAS-STING pathway is regulated by LLPS in two ways.

On the one hand, cGAS can form a membrane-free cellular compartment through phase separation. cGAS has two structural domains: the C-terminal nucleotidyltransferase (NTase) domain and the non-fixed, positively charged N-terminal. These domains can induce LLPS by promoting the binding of cGAS to dsDNA through multivalent interactions (97). High concentrations of cGAS-DNA complexes can increase the activity of cGAS, promoting the synthesis of cGAMP.

**Figure 2:**
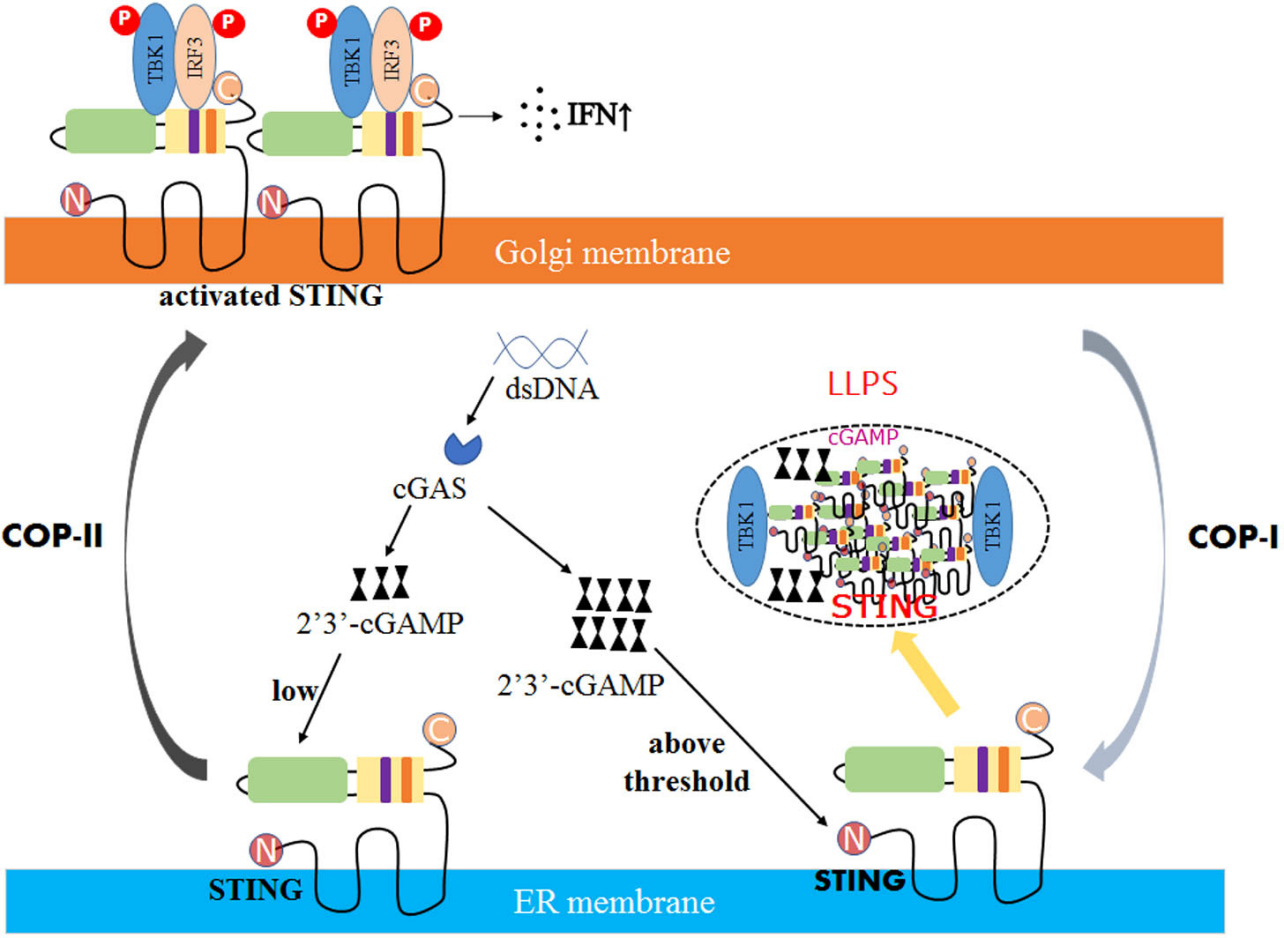
The mechanism of STING phase separators interferes with innate immune signaling. In DNA virus-infected cells, the recognition of double-stranded DNA (dsDNA) by STING on the ER membrane is followed by activating the second messenger 2′3′-cGAMP generated by the DNA sensor cGAS. Due to low levels of 2′3′-cGAMP, STING is transferred to the Golgi apparatus, polymerized, and recruited to activate TBK1 and IRF3, promoting the production of cytokines such as type I interferon (IFN). However, when the concentration of 2′3′-cGAMP reaches a threshold, the STING phase separator forms, recruiting cGAMP and unphosphorylated TBK1 into liquid droplets, separating STING and TBK1 from its downstream signaling and preventing the overactivation of the innate immune response.

On the other hand, viruses can also induce the formation of STING phase-separators through LLPS. Excess 2’3’-cGAMP was discovered to enable STING to form biomolecular agglutination in DNA virus-infected cells (98). It recruits excess intracellular STING and TBK1, but not IRF3, thereby inhibiting the phosphorylation of IRF3 and preventing overactivation of the cGAS-STING pathway (**Figure 2**) (99).

In addition, antiviral sensors isolated in IBs can suppress the activation of downstream pathways (50). Take the example of IBs formed by RSV. Initially, MAVS can activate cytosolic kinase IKK and TBK1 to activate transcription factors NF-κB and IRF3 (100). Lifland et al. found that MAVS and MDA5 are localized in IBs. Importantly, the proximity ligation assay showed that RSV N proteins co-localize with MAVS and MDA5 in IBs (101). Consequently, sequestering MAVS and MDA5 into IBs leads to the strong inhibition of type I interferon production. Furthermore, Fatoumatta et al. observed that p65 recruited into IBs can block the NF-κB signaling pathway (53).

## Conclusion

The role of phase separation in viral adsorption, replication, assembly, and release is a hot topic. Biomolecular condensates formed through LLPS can concentrate replication machinery, facilitate viral gene transcription and expression, and regulate innate immune responses by constraining the host sensors in IBs. As a result, phase separation offers a new perspective on viral replication, assembly, and egress within the host cell and a promising treatment strategy for viral infections.

However, the underlying mechanisms of LLPS’s formation and action during viral replication, capsid assembly, progeny egress, and antiviral immune regulation remain unknown. All macromolecules can form a network through multivalent interaction, resulting in phase separation. The intracellular environment, on the other hand, is harsh. How do viral proteins recognize and interact before aggregating at a certain concentration? How do viruses maintain the right balance of LLPS assembly and disassembly to meet the needs of viral replication during the infection? The phase separation phenomenon has only been observed in RNA viruses; however, it is unknown whether phase separation occurs during most other DNA viral infections and HSV-1 and EBV.

Moreover, the proteins’ multivalency is indispensable for forming phase separation. Proteins containing intrinsically disordered regions (IDRs) or SRC homology 3 (SH3) repeats can interact, forming condensates. Viral proteins, like HSV-1 UL11 and ICP4, have IDRs, which can drive the formation of condensates. In addition to IDRs, other modules, like SH3 repeats, and multiple proline-rich motifs (PRM) repeats, are also involved in the multivalent interactions between proteins, leading to the formation of multimers. The formation of multimers can drive the formation of LLPS. Additionally, SH3 repeats and PRM repeats can usually be found in eukaryotic protein. So, it can be presumed that viral proteins may not have these modules and cannot mediate the formation of LLPS through these modules.

Furthermore, the investigation of LLPS’s roles in antiviral immune regulation, such as the cGAS-STING signaling pathway, is still early. It is unclear whether LLPS is involved in other immune response processes like the oligomerization of the cytosolic viral RNA sensors RIG-I and MDA5 and the formation of the inflammasome. More exciting findings on the roles of LLPS in immune regulation are expected to emerge. Overall, LLPS provides a solid theoretical foundation for understanding viral infection. Further investigations into the underlying mechanism of LLPS formation and its roles during viral infections will aid in developing novel antiviral therapies.

## Author contributions

WW designed and supervised the manuscript. LB and WW wrote the preliminary draft manuscript. WW, BY, WM, and HW reviewed the preliminary draft manuscript. JZ did the analyses. CZ, FS, and WW edited, revised, and finalized the manuscript. All the authors read and approved the manuscript.

## Funding

This study was supported by the Natural Science Foundation of China (32072838).

## Conflict of interest

The authors declare that the research was conducted in the absence of any commercial or financial relationships that could be construed as a potential conflict of interest.

## Publisher’s note

All claims expressed in this article are solely those of the authors and do not necessarily represent those of their affiliated organizations, or those of the publisher, the editors and the reviewers. Any product that may be evaluated in this article, or claim that may be made by its manufacturer, is not guaranteed or endorsed by the publisher.

## Glossary

**Table:**

LLPS	liquid-liquid phase separation
MLO	membraneless organelle
SG	stress granule
RC	replication compartment
IB	inclusion of body
SARS-COV-2	severe acute respiratory syndrome coronavirus
2RABV	rabies virus
vRNA	viral RNA
IDRs	intrinsically disordered regions
PLDs	prion-like domains
LCDs	low-complexity sequence domains
IDPs	intrinsically disordered proteins
SH3	SRC homology 3
PRM	proline-rich motif
gRNA	genomic RNA
PrLD	prion-like disordered protein domain
HIV-1	human immunodeficiency virus type 1
ZnF	zinc finger
HSV-1	herpes simplex virus type
1EBV	Epstein Barr virus
RdRp	RNA-dependent RNA polymerase
ERGIC	ER-Golgi intermediate
Compartment	
MA	matrix
CA	capsid
NC	nucleocapsid
STING	stimulator of interferon gene
cGAMP	cyclic GMP- AMP
